# Trainee Physician Milestone Ratings and Patient Complaints in Early Posttraining Practice

**DOI:** 10.1001/jamanetworkopen.2023.7588

**Published:** 2023-04-11

**Authors:** Misop Han, Stanley J. Hamstra, Sean O. Hogan, Eric Holmboe, Kelly Harris, Eric Wallen, Gerald Hickson, Kyla P. Terhune, Donald W. Brady, Bruce Trock, Kenji Yamazaki, Jessica L. Bienstock, Henry J. Domenico, William O. Cooper

**Affiliations:** 1 Johns Hopkins University School of Medicine, Baltimore, Maryland; 2 University of Toronto, Toronto, Ontario, Canada; 3 Accreditation Council for Graduate Medical Education, Chicago, Illinois; 4 Northwestern University, Chicago, Illinois; 5 Children’s Hospital of Colorado, Aurora; 6 University of North Carolina, Chapel Hill; 7 Vanderbilt University Medical Center, Nashville, Tennessee

## Abstract

**Importance:**

Evaluation of trainees in graduate medical education training programs using Milestones has been in place since 2013. It is not known whether trainees who have lower ratings during the last year of training go on to have concerns related to interactions with patients in posttraining practice.

**Objective:**

To investigate the association between resident Milestone ratings and posttraining patient complaints.

**Design, Setting, and Participants:**

This retrospective cohort study included physicians who completed Accreditation Council for Graduate Medical Education (ACGME)–accredited programs between July 1, 2015, and June 30, 2019, and worked at a site that participated in the national Patient Advocacy Reporting System (PARS) program for at least 1 year. Milestone ratings from ACGME training programs and patient complaint data from PARS were collected. Data analysis was conducted from March 2022 to February 2023.

**Exposures:**

Lowest professionalism (P) and interpersonal and communication skills (ICS) Milestones ratings 6 months prior to the end of training.

**Main Outcomes and Measures:**

PARS year 1 index scores, based on recency and severity of complaints.

**Results:**

The cohort included 9340 physicians with median (IQR) age of 33 (31-35) years; 4516 (48.4%) were women physicians. Overall, 7001 (75.0%) had a PARS year 1 index score of 0, 2023 (21.7%) had a score of 1 to 20 (moderate), and 316 (3.4%) had a score of 21 or greater (high). Among physicians in the lowest Milestones group, 34 of 716 (4.7%) had high PARS year 1 index scores, while 105 of 3617 (2.9%) with Milestone ratings of 4.0 (proficient), had high PARS year 1 index scores. In a multivariable ordinal regression model, physicians in the 2 lowest Milestones rating groups (0-2.5 and 3.0-3.5) were statistically significantly more likely to have higher PARS year 1 index scores than the reference group with Milestones ratings of 4.0 (0-2.5 group: odds ratio, 1.2 [95% CI, 1.0-1.5]; 3.0-3.5 group: odds ratio, 1.2 [95% CI, 1.1-1.3]).

**Conclusions and Relevance:**

In this study, trainees with low Milestone ratings in P and ICS near the end of residency were at increased risk for patient complaints in their early posttraining independent physician practice. Trainees with lower Milestone ratings in P and ICS may need more support during graduate medical education training or in the early part of their posttraining practice career.

## Key Points

**Question:**

Do trainee physicians with lower Milestones ratings in professionalism (P) and interpersonal and communication skills (ICS) during the last year of training receive more patient complaints in early posttraining practice?

**Findings:**

In this cohort study of 9340 physicians, those with Milestone ratings in P and ICS below the graduation target during training were statistically significantly more likely to have patient complaints in the first year of posttraining physician practice.

**Meaning:**

In this study, lower Milestone ratings in P and ICS were associated with higher risk of patient complaints, suggesting that these individuals may need more support during graduate medical education training or in the early part of their posttraining practice career.

## Teaser

This cohort study investigates the association of resident Milestone ratings in professionalism and interpersonal and communication skills with posttraining patient complaints.

## Introduction

The Accreditation Council for Graduate Medical Education (ACGME) Milestones, implemented in 2013 and now required for all ACGME-accredited training programs, are competency-based behavioral descriptions of performance designed to help programs support the developmental progression of trainees during graduate medical education (GME).^1,2^ Milestones ratings provide a framework for evaluating performance in core competencies (medical knowledge [MK], patient care [PC], practice-based learning and improvement, systems-based practice, interpersonal and communication skills [ICS], and professionalism [P]).^1^ Each specialty uses a unique set of subcompetencies for each core competency, with considerable conceptual overlap in Milestones across specialties.^3^ While nearly every trainee in ACGME-accredited training programs currently receives Milestones ratings,^4^ it is not known whether Milestone ratings are associated with physician performance once individuals enter posttraining practice.

Physicians in the early years of posttraining practice are at the greatest risk for unsolicited patient complaints^5^ and medical malpractice lawsuits.^6,7^ The Patient Advocacy Reporting System (PARS), a reporting and intervention system developed at Vanderbilt Center for Patient and Professional Advocacy (CPPA), is based on unsolicited patient complaints and is designed to alert physicians to their risk of adverse outcomes, malpractice claims, and well-being concerns.^8,9,10,11,12,13^ PARS patient complaints capture a broad range of issues related to communication, concern for patients and families, care and treatment, access and availability, environment, and billing.^8,9,10,11,12,13^ Many health care institutions and physician practices currently use PARS to monitor and mitigate their physicians’ professionalism and interpersonal communication performance concerns as described in patient complaints.^14^

In this study, we linked ACGME Milestone ratings in P and ICS during the last year of GME with PARS patient complaints during the first year of posttraining practice. We aimed to investigate the association between resident evaluation measures and posttraining performance.

## Methods

### Study Design

We conducted a retrospective cohort study of trainees in ACGME-accredited programs who completed training during the study period (July 1, 2015, to June 30, 2019). The study was reviewed and approved by institutional review boards at Vanderbilt University Medical Center and the ACGME. Because the analysis was conducted on data sets that were constructed in a way that it was not possible to link the data set to an individual, the institutional review boards determined that the study met 45 CFR 46.104 (d) category (4ii) for exempt review; therefore, the requirement for informed consent was waived. The study followed the Strengthening the Reporting of Observational Studies in Epidemiology (STROBE) reporting guideline for conducting cohort studies.^15^

### Data Sources

Data for the study were obtained from 2 sources: ACGME Milestone ratings and PARS data. Milestone ratings are reported to the ACGME biannually on more than 150 000 residents and fellows in ACGME-accredited training programs and include unique individual identifiers, details about the training program, specialty, completion year, and Milestone ratings for all subcompetencies. PARS data include unsolicited complaints for each physician and an annual PARS score based on the recency and severity of patient complaints.^8,14^ Data for more than 200 hospitals and multispecialty practices throughout the United States are collected under individual contracts with each participating site, which allow for use of aggregated, deidentified data for research. The PARS program currently has approximately 70 000 physicians in its database, which represents approximately 5% of practicing US physicians. The 2 data sets were linked and then deidentified by a computer systems analyst employed by Vanderbilt University Medical Center who was not involved in the conduct of the analyses using National Provider Identifier numbers, name, date of birth, and publicly available training program information (eg, posttraining practice websites) (eFigure in Supplement 1).^5^

### Study Participants

We identified all residents who completed training during the study period. If a trainee completed 2 programs (eg, subspecialty fellowship after specialty residency) during the study period, we chose the last training program completed. To facilitate identification of PARS outcomes, we required that cohort members have at least 1 year of posttraining practice at a PARS site. Trainees in disciplines with limited direct patient contact, including pathology and diagnostic radiology, were excluded based on prior work.^14^ Study follow-up for outcomes began on the person’s first day of employment and ended 365 days after the first day of employment at their posttraining health care organization.

### Exposure: Lowest Milestone Ratings

Each specialty in the ACGME Milestones data set has created its own specific subcompetencies and Milestones for each core competency.^3^ While the number of subcompetencies and specific language differs across specialties, there are common themes in the Milestones language for ratings across specialties.^3^ Milestone ratings are based on the 5 developmental levels of the Dreyfus 5-stage model of adult skill acquisition, in which level 1 equates to novice; level 2, advanced beginner; level 3, competent; level 4, proficient; and level 5, progress toward expertise.^16^ Level 4 is the recommended developmental target for a trainee at the completion of training.^17,18^ The primary exposure of interest was the lowest of either the P or ICS ratings at the assessment 6 months prior to the end of residency training. Secondary exposures included the lowest subcompetency ratings for PC and MK subcompetencies, as these may be related to patient complaints about the care received and/or how the plan of care was communicated (eg, PC: “Dr. ____ put the wrong medication on my prescription. The pharmacist caught it, but…”; MK: “Dr. ___ was not aware of changes in the staging of breast cancer from a year ago.”)

### Study Outcome: PARS Patient Complaint Scores in Early Practice

In PARS, each health care institution has a system to receive and record unsolicited patient complaint reports and for secure transfer of their complaint database to CPPA. PARS methods are then used to code unsolicited patient complaints using a validated coding system with high interrater and test-retest reliabilities.^14,19^ Complaint codes include 34 specific categories subsumed under 6 general categories including communication, concern for patients and families, care and treatment, access and availability, environment, and billing.

The PARS index score for each physician is typically calculated based on a rolling 4 years of unsolicited patient complaints, with more recent and more severe complaints weighted more than older and less severe complaints.^14^ We used the PARS year 1 index score, also weighted for recency and severity, to identify early career patient complaints. The PARS year 1 index for the study sample ranged from 0 to 88, with higher scores indicating more recent and severe complaints. We grouped PARS year 1 index scores to reflect low (0), moderate (1-20), and high (≥21) risk of malpractice claims and surgical complications, which paralleled groupings for previous studies.^8,10^

### Covariates and Confounding Variables

We identified covariates a priori as those variables likely to be confounders, including medical vs surgical specialty,^8^ gender, age, year of completing training, years since medical school completion, graduation from a non-US medical school, and PARS site.^5,14,20^ We also included the size of the training program and the region of the country where the training program was located to capture potential differences in Milestones scoring and differences in trainees across program types, which we hypothesized could also be associated with the primary outcome of patient complaints.

### Statistical Analysis

We calculated the lowest Milestone rating for P or ICS as the primary exposure variable. Based on the distribution of the Milestone ratings, we grouped lowest Milestone ratings into the following ordinal categories related to the developmental progression of the trainee: level 1 or 2 (novice or beginner), 0 to 2.5; level 3 (competent), 3.0 to 3.5; level 4 (proficient), 4.0; and level 5 (expert), 4.5 to 5.0. A score of 4.0 served as the reference for each analysis because it represented the recommended graduation target.^16^ We summarized the distribution of covariates across each of the primary exposure categories using counts and percentages for categorical variables and medians with IQRs for continuous variables. To test the association of Milestone categories with PARS year 1 index score, we calculated odds ratios (ORs), 95% CIs, and *P* values using a cumulative link mixed-effects model (ordinal regression model with mixed effects) with outcome of PARS year 1 index score category. ORs showed the odds of clinicians being included in a higher year 1 PARS index score category compared with clinicians in Milestone level 4 (proficient group). Models were adjusted for clinician gender, age, training year, US medical school status, academic vs nonacademic practice setting, surgical vs nonsurgical specialty, and program size decile (fixed effects) and included a random effect for PARS site. A Brant-Wald test was used to test the appropriateness of the proportional odds assumption for Milestone category.

To test the robustness of study assumptions, we performed several sensitivity analyses, including separate models adjusting for training program specialty (eg, anesthesiology, internal medicine, pediatrics, general surgery, neurology) as a fixed effect, accounting for clustering on (1) residency site, (2) training program specialty, (3) training program subspecialty (eg, adult rheumatology, pediatric rheumatology), and (4) program size decile. Analyses were conducted using R version 4.2.1 (R Foundation for Statistical Computing). A 2-sided *P* < .05 was used as a cutoff for statistical significance.

## Results

The final study cohort included 9340 physicians (median [IQR] age, 33 [31-35] years). Nearly half of the study cohort were women physicians (4516 [48.4%]), and cohort members completed training in all 4 regions of the United States (Table). Posttraining practice locations were more commonly in the Midwest and West regions. Less than 20% of cohort members graduated from non-US medical schools (1712 [18.3%]), two-thirds entered posttraining practice in academic settings (6230 [66.7%]), and 1 in 5 entered posttraining practice in surgical specialties (2042 [21.9%]). There was a smaller number of cohort members who completed training in 2015, reflecting the incremental adoption of Milestones by specialties over time.

**Table.** Characteristics of the Cohort by Minimum Professionalism and Interpersonal and Communication Skills Milestone Categories

Characteristic	Participants by minimum professionalism and interpersonal and communication skills Milestone categories at the penultimate rating period prior to completion of training, No. (%)					
	Novice or beginner (score 0-2.5) (n = 716)	Competent (score 3.0-3.5) (n = 3853)	Proficient (score 4.0) (n = 3617)	Expert (score 4.5-5.0) (n = 1154)	Total (N = 9340)	*P* value^a^
Age, median (IQR), y	33 (31-35)	32 (31-35)	33 (31-35)	33 (32-35)	33 (31-35)	<.001
Clinician gender						
Women	322 (45.0)	1897 (49.2)	1763 (48.7)	534 (46.3)	4516 (48.4)	.09
Men	393 (54.9)	1955 (50.7)	1853 (51.2)	619 (53.6)	4820 (51.6)	
Missing	1 (0.1)	1 (<0.1)	1 (<0.1)	1 (0.1)	4 (<0.1)	
Year of training						
2015	27 (3.8)	64 (1.7)	65 (1.8)	23 (2.0)	179 (1.9)	<.001
2016	176 (24.6)	957 (24.8)	834 (23.1)	225 (19.5)	2192 (23.5)	
2017	163 (22.8)	965 (25.0)	924 (25.5)	277 (24.0)	2329 (24.9)	
2018	193 (27.0)	1001 (26.0)	942 (26.0)	331 (28.7)	2467 (26.4)	
2019	157 (21.9)	866 (22.5)	852 (23.6)	298 (25.8)	2173 (23.3)	
Program region						
Midwest	247 (34.5)	1096 (28.4)	966 (26.7)	275 (23.8)	2584 (27.7)	<.001
Northeast	130 (18.2)	733 (19.0)	758 (21.0)	289 (25.0)	1910 (20.4)	
South	193 (27.0)	938 (24.3)	895 (24.7)	287 (24.9)	2313 (24.8)	
West	146 (20.4)	1084 (28.1)	998 (27.6)	302 (26.2)	2530 (27.1)	
Puerto Rico	0	2 (0.1)	0	1 (0.1)	3 (<0.1)	
PARS site region						
Midwest	287 (40.1)	1235 (32.1)	1114 (30.8)	327 (28.3)	2963 (31.7)	<.001
Northeast	63 (8.8)	340 (8.8)	372 (10.3)	152 (13.2)	927 (9.9)	
South	177 (24.7)	887 (23.0)	831 (23.0)	279 (24.2)	2174 (23.3)	
West	189 (26.4)	1391 (36.1)	1300 (35.9)	396 (34.3)	3276 (35.1)	
US vs non-US medical school						
Non-US medical school	154 (21.5)	793 (20.6)	601 (16.6)	164 (14.2)	1712 (18.3)	<.001
US medical school	562 (78.5)	3060 (79.4)	3016 (83.4)	990 (85.8)	7628 (81.7)	
Academic vs nonacademic practice setting						
Academic	421 (58.8)	2488 (64.6)	2480 (68.6)	841 (72.9)	6230 (66.7)	<.001
Nonacademic	295 (41.2)	1365 (35.4)	1137 (31.4)	313 (27.1)	3110 (33.3)	
Surgical vs nonsurgical clinician						
Nonsurgical	579 (80.9)	3244 (84.2)	2726 (75.4)	749 (64.9)	7298 (78.1)	<.001
Surgical	137 (19.1)	609 (15.8)	891 (24.6)	405 (35.1)	2042 (21.9)	

Abbreviation: PARS, Patient Advocacy Reporting System.

^a^ *P* values calculated using a Mann-Whitney *U* test for continuous variables and Pearson χ^2^ test for categorical variables.

Less than 10% (716 [7.7%]) received ratings for P and ICS competencies in the lowest group (0-2.5) during the last year of training, and these physicians were comparable with the other groups in terms of age, gender, and year of training completion (Table). There was no difference by gender in the proportion of physicians with the lowest Milestones ratings. Physicians with Milestones ratings below graduation target (ie, <4.0) were more likely to graduate and enter posttraining practice in the Midwest region of the United States, more likely to enter posttraining practice in a nonacademic setting, and more likely to practice in a nonsurgical specialty.

**Figure 1.**
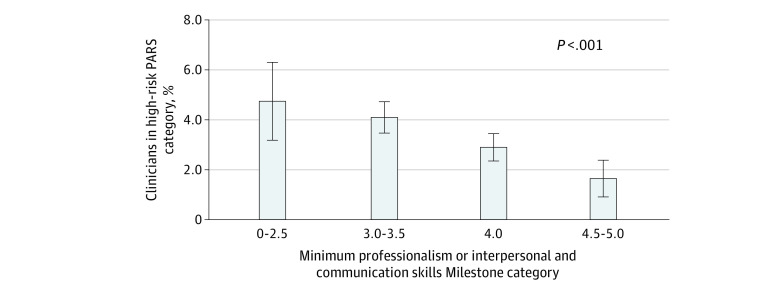
Proportion of Clinicians with Patient Advocacy Reporting System (PARS) Year 1 Index Score of 21 or Greater by Minimum Professionalism and Interpersonal and Communication Skills Milestone Category During the Last Year of Residency Training

Overall, 7001 (75.0%) had a PARS year 1 index score of 0, 2023 (21.7%) had a PARS year 1 index score of 1 to 20, and 316 (3.4%) had a PARS year 1 index score of 21 or greater (eTable 1 in Supplement 1). Outcomes did not differ by gender; 1116 of 4516 women (24.7%) and 1223 of 4820 men (25.4%) had a year 1 PARS index score greater than 0 (*P* = .21). Among physicians in the lowest Milestones group (ratings 0-2.5), 34 of 716 (4.7%) had high PARS year 1 index scores, while among those with ratings of 3.0 to 3.5, 158 of 3853 (4.1%) had high PARS scores (Figure 1). Among the group with Milestones ratings of 4.0 (proficient), 105 of 3617 (2.9%) had high PARS year 1 index scores, while those with the highest Milestones ratings had the lowest PARS Year 1 Index Scores (19 of 1154 [1.6%]).

**Figure 2.**
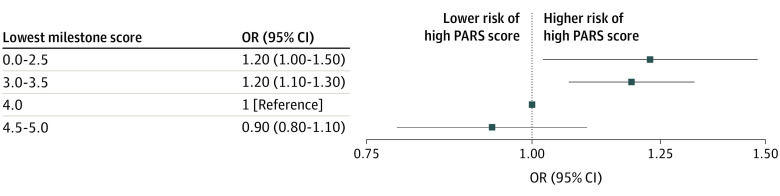
Adjusted Association of Professionalism and Interpersonal and Communication Skills Milestone Category With Patient Advocacy Reporting System (PARS) Year 1 Index Score Category OR indicates odds ratio.

**Figure 3.**
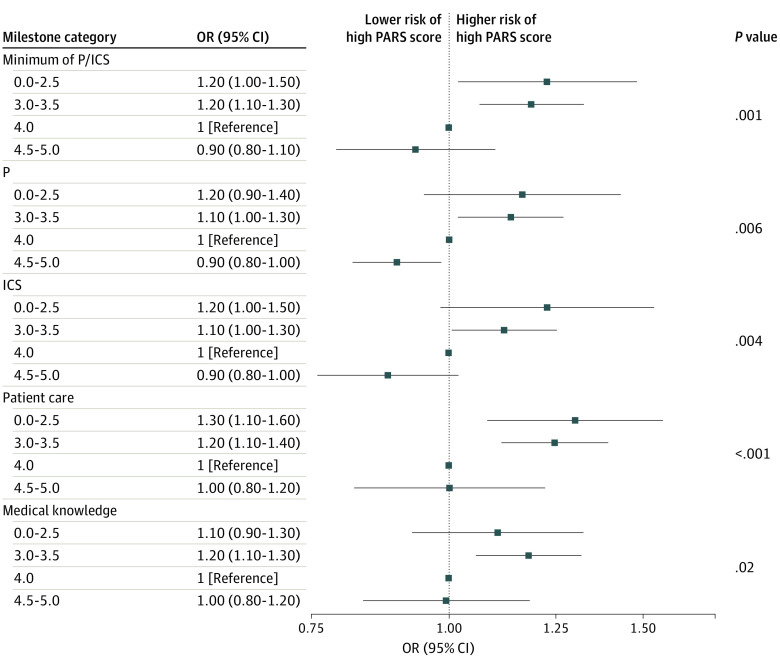
Adjusted Association of Milestone Category With Patient Advocacy Reporting System (PARS) Year 1 Index Score Category ICS indicates interpersonal and communication skills; OR, odds ratio; P, professionalism.

In the primary multivariable ordinal regression model, physicians in the lowest Milestone rating groups (level 1 or 2 and 3) were statistically significantly more likely to have higher PARS year 1 index scores than the reference group of level 4, proficient (level 1 or 2: OR, 1.2 [95% CI, 1.0-1.5]; level 3: OR, 1.2 [95% CI, 1.1-1.3]) (Figure 2). The Brant-Wald test showed no violation of the proportional odds assumption in the primary model. Similar associations were seen when comparisons were made across individual Milestone ratings for other competencies, although the association between MK Milestones and PARS outcomes was less strong (Figure 3). Full statistical models are shown in eTables 2 to 6 in Supplement 1.

**Figure 4.**
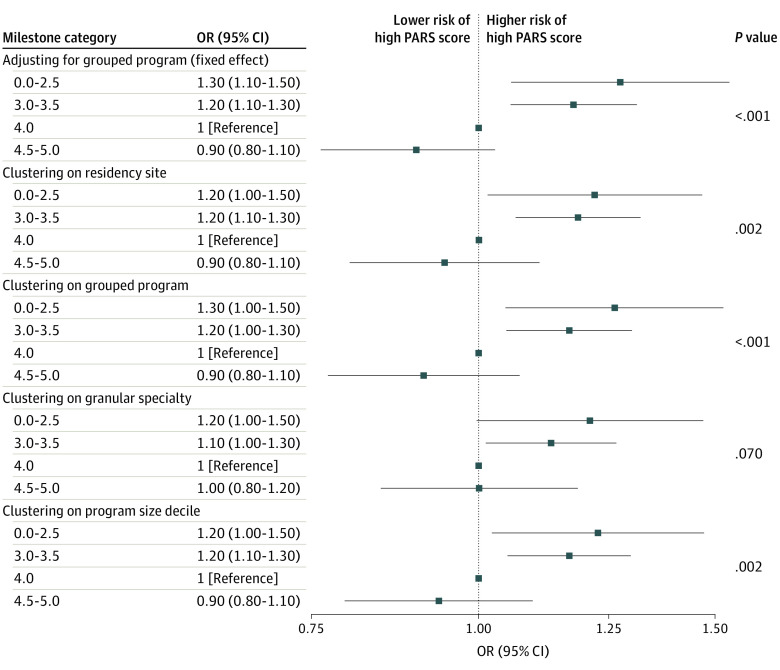
Adjusted Association of Milestone Category With Patient Advocacy Reporting System (PARS) Year 1 Index Score Category Using Alternate Clustering Strategies OR indicates odds ratio.

Sensitivity analyses testing the robustness of study assumptions considered several variables as fixed and random effects, including models that accounted for clustering on residency site, training program, granular specialty variables, and program size deciles (Figure 4). The results of these sensitivity analyses did not differ in material ways from the primary analyses. Full models for the sensitivity analyses are shown in eTables 7 to 11 in Supplement 1.

## Discussion

In this cohort study of 9340 physicians, we linked ACGME Milestone ratings in P and ICS during trainees’ last year of GME and their unsolicited patient complaints as measured by the PARS program during the first year of posttraining physician practice. There are several important findings in this study. First, a small proportion of trainees (<10%) received the lowest Milestones ratings in P and ICS in their last year of GME training. Second, physicians in the lowest Milestone group were 1.20 times more likely to be in the highest risk PARS category (≥21) compared with physicians in the level 4 group (reference group). Third, graduating trainees who had proficient ratings had similar patient complaints as those who had expert ratings. These findings were robust to several sensitivity analyses designed to test key study assumptions.

Prior work by Papadakis and others^21,22,23^ showed the association between performance during medical school and subsequent disciplinary action by medical boards. Graduating trainees with low professionalism ratings during training were also more likely to experience severe disciplinary action later in their careers.^24^ A recent study with a cohort of pulmonary medicine trainees showed that low Milestones ratings in P and ICS during internal medicine residency were associated with low Milestones ratings in pulmonary fellowship.^25^ This study builds on this previous body of work by providing a direct association between ratings of competency during training and patient complaints following graduation. Because higher PARS scores are associated with poor patient outcomes,^9,10^ concerns for clinician well-being,^11,26^ and a physician’s risk for litigation,^8^ PARS scores are important measures of physicians in posttraining practice and may provide an earlier warning of problems that could worsen to more severe outcomes if not addressed.

The findings of this study have several important implications, including evidence that resident evaluations using Milestones ratings are associated with an independently obtained performance metric in the posttraining workplace. While the desired process during residency training includes sharing of Milestones data at regular intervals to improve awareness around professionalism and other competencies, there are times when this does not occur. The findings of this study highlight the importance of regular developmental assessment and sharing of progress. GME faculty could use Milestones ratings during training to identify trainees whose performance is below target and intervene or even extend training. For individual trainees performing below target in the last year of training, the findings of this study could be used to make them aware of their performance and encourage the trainee to seek resources such as coaching or to at least seek feedback at an earlier date than might typically happen when they enter posttraining practice. Early career physicians may also want to use Milestones data as they develop formal or informal individualized learning plans as part of their lifelong learning.^27,28^

The findings also suggest that an approach to “pass along” trainees who underperform in competencies relevant to professionalism puts physicians early in their posttraining practice at risk for patient complaints. Future studies might assess whether improvement in Milestone ratings, either self-initiated or as a result of an intervention, results in fewer patient complaints in posttraining practice. If improvement in Milestones is associated with improved posttraining performance, additional efforts in GME should be made for all trainees to meet recommended targets. Other future studies might assess the outcomes of efforts to harmonize Milestones ratings across specialties on the associations described in this study.^29^

### Limitations

The study has several potential limitations that should be considered in interpreting the results. Patient complaints, which describe a range of behaviors, may only represent a portion of a physician’s professionalism. Several sources of bias might affect the association between Milestones ratings and posttraining practice performance. For example, the idea of leniency bias, while not demonstrated statistically, could result in trainees who have true concerns related to professionalism being identified through the Milestones as high performers, which would bias our findings toward the null. In fact, there were some cohort members who received ratings at or above proficiency who were associated with a high number of patient complaints. To reduce the impact of this limitation, we used the penultimate Milestones rating, which could be less subject to leniency bias.

It is also possible that disparities and potential biases related to gender and race and ethnicity might have affected Milestones ratings and/or patient complaints.^30,31,32,33^ In the present study, we found no difference in the proportion rated as below target for the Milestones by gender and no difference in the proportion with patient complaints by gender. Because the ACGME data during the study period did not have reliable race and ethnicity data, we were not able to include these variables in the current study. Nonetheless, the possibility of bias does suggest the need for further study.

In addition, while we focused on P and ICS, performance in other competencies might interrelate in different ways for individuals. Some physicians might have isolated professionalism performance concerns, while others may be viewed as professional but demonstrate clinical or functional incompetence. Others might have challenges across multiple domains. Future studies could explore whether specific complaint types (eg, communication, concern for patients and families, care, and treatment) might map to specific Milestones.

The study population includes approximately 6% of graduating residents during the study period. In addition, there was a small number of residents who were excluded because they changed jobs within the first year. While the study group is generally comparable with the overall ACGME graduating population (eg, 48% women in the study and 46% in the overall ACGME population; 22% vs 20% surgical; 18% vs 22% international medical graduate),^34^ there may be differences between the study population and the overall population of physicians that limit the generalizability of the study findings, including practice site.

## Conclusions

In this study, low P and ICS Milestone ratings near the end of residency training were associated with greater numbers of patient complaints in physicians’ early posttraining practice. Trainees with lower Milestone ratings in P/ICS may need more support during graduate medical education training or in the early part of their post-training practice career.

## Notes

Supplement 1. **eFigure.** Matching Process Linking ACGME Data to PARS Data **eTable 1.** Year 1 PARS Score Category **eTable 2.** Cumulative Link Mixed-Effects Model Testing Association of Minimum Professionalism/Communication Milestone Category With Year 1 PARS Category **eTable 3.** Cumulative Link Mixed-Effects Model Testing Association of Lowest Professionalism Milestone Category With PARS Year 1 Index Score Category **eTable 4.** Cumulative Link Mixed-Effects Model Testing Association of Lowest Communication Milestone Category With PARS Year 1 Index Score Category **eTable 5.** Cumulative Link Mixed-Effects Model Testing Association of Lowest Patient Care Milestone Category With PARS Year 1 Index Score Category **eTable 6.** Cumulative Link Mixed-Effects Model Testing Association of Lowest Medical Knowledge Milestone Category With PARS Year 1 Index Score Category **eTable 7.** Sensitivity Analysis: Ordinal Regression Model Treating Program as a Fixed Effect **eTable 8.** Sensitivity Analysis: Cumulative Link Mixed-Effects Model Clustering on Residency Site **eTable 9.** Sensitivity Analysis: Cumulative Link Mixed-Effects Model Clustering on Training Program **eTable 10.** Sensitivity Analysis: Cumulative Link Mixed-Effects Model Clustering on Clinician Specialty **eTable 11.** Sensitivity Analysis: Cumulative Link Mixed-Effects Model Clustering on Program Size Decile

Supplement 2.
Data Sharing Statement
