# Supplementary material for: Trainee Physician Milestone Ratings and Patient Complaints in Early Posttraining Practice
Source: JAMA Netw Open. 2023 Apr 11;6(4):e237588. doi: 10.1001/jamanetworkopen.2023.7588 (PMC10091163; doi:10.1001/jamanetworkopen.2023.7588)
Supplement: Supplement 1. — eFigure. Matching Process Linking ACGME Data to PARS Data eTable 1. Year 1 PARS Score Category eTable 2. Cumulative Link Mixed-Effects Model Testing Association of Minimum Professionalism/Communication Milestone Category With Year 1 PARS Category eTable 3. Cumulative Link Mixed-Effects Model Testing Association of Lowest Professionalism Milestone Category With PARS Year 1 Index Score Category eTable 4. Cumulative Link Mixed-Effects Model Testing Association of Lowest Communication Milestone Category With PARS Year 1 Index Score Category eTable 5. Cumulative Link Mixed-Effects Model Testing Association of Lowest Patient Care Milestone Category With PARS Year 1 Index Score Category eTable 6. Cumulative Link Mixed-Effects Model Testing Association of Lowest Medical Knowledge Milestone Category With PARS Year 1 Index Score Category eTable 7. Sensitivity Analysis: Ordinal Regression Model Treating Program as a Fixed Effect eTable 8. Sensitivity Analysis: Cumulative Link Mixed-Effects Model Clustering on Residency Site eTable 9. Sensitivity Analysis: Cumulative Link Mixed-Effects Model Clustering on Training Program eTable 10. Sensitivity Analysis: Cumulative Link Mixed-Effects Model Clustering on Clinician Specialty eTable 11. Sensitivity Analysis: Cumulative Link Mixed-Effects Model Clustering on Program Size Decile [file jamanetwopen-e237588-s001.pdf]

Supplemental Online Content

Han M, Hamstra SJ, Hogan SO, et al. Trainee physician Milestone ratings and patient complaints in early posttraining practice. *JAMA Netw Open.* 2023;6(4):e237588. doi:10.1001/jamanetworkopen.2023.7588

eFigure. Matching Process Linking ACGME Data to PARS Data

eTable 1. Year 1 PARS Score Category

eTable 2. Cumulative Link Mixed-Effects Model Testing Association of Minimum Professionalism/Communication Milestone Category With Year 1 PARS Category

eTable 3. Cumulative Link Mixed-Effects Model Testing Association of Lowest Professionalism Milestone Category With PARS Year 1 Index Score Category

eTable 4. Cumulative Link Mixed-Effects Model Testing Association of Lowest Communication Milestone Category With PARS Year 1 Index Score Category

eTable 5. Cumulative Link Mixed-Effects Model Testing Association of Lowest Patient Care Milestone Category With PARS Year 1 Index Score Category

eTable 6. Cumulative Link Mixed-Effects Model Testing Association of Lowest Medical Knowledge Milestone Category With PARS Year 1 Index Score Category

eTable 7. Sensitivity Analysis: Ordinal Regression Model Treating Program as a Fixed Effect

eTable 8. Sensitivity Analysis: Cumulative Link Mixed-Effects Model Clustering on Residency Site

eTable 9. Sensitivity Analysis: Cumulative Link Mixed-Effects Model Clustering on Training Program

eTable 10. Sensitivity Analysis: Cumulative Link Mixed-Effects Model Clustering on Clinician Specialty

eTable 11. Sensitivity Analysis: Cumulative Link Mixed-Effects Model Clustering on Program Size Decile

This supplemental material has been provided by the authors to give readers additional information about their work.

© 2023 Han M et al. *JAMA Network Open.*

eFigure. Matching Process Linking ACGME Data to PARS Data

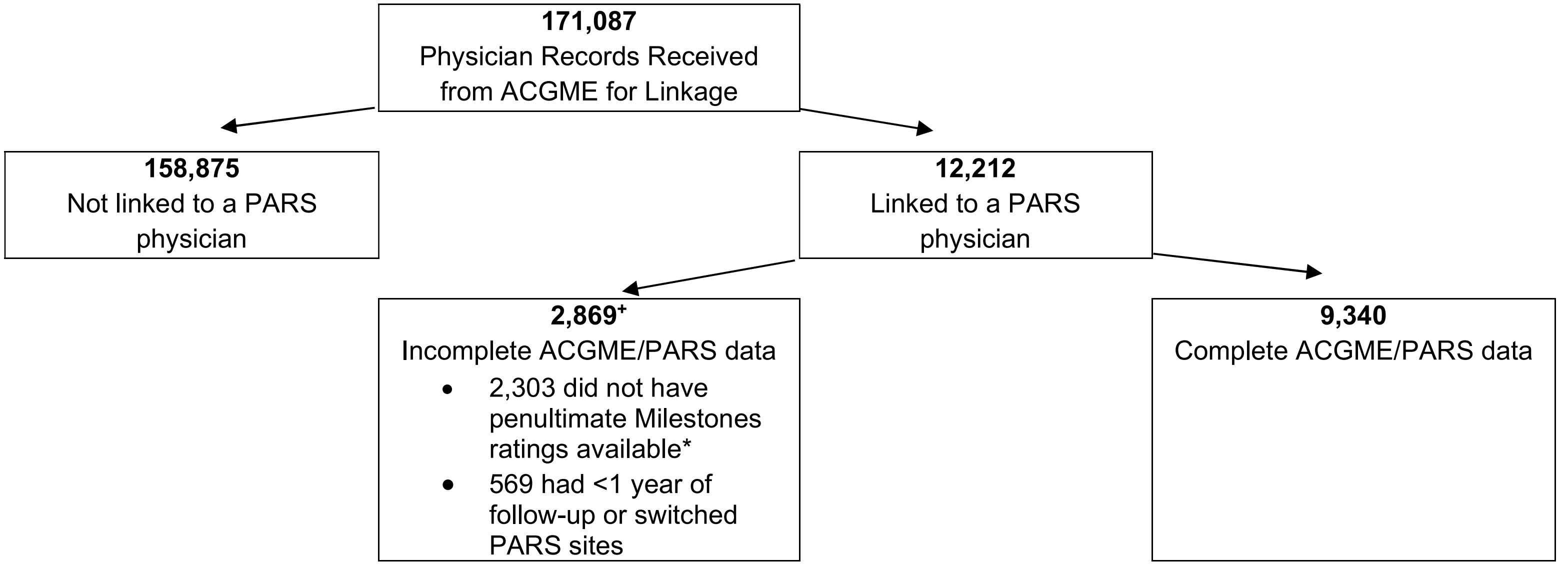

*****_2,066 completed training before Milestones available for their specialty; 237 did not enter practice within a year or had Milestones data earlier in residency but not at the penultimate rating period._

[+]_Comparison of 2689 excluded physicians to the final study cohort:_

	**Final Study Cohort (N=9340)**	**Not Included (N=2869)**
**Gender; N (%)**		
Female	4516 (48.4%)	1333 (46.5%)
Male	4820 (51.6%)	1534 (53.5%)
Missing	4 (0.0%)	2 (0.1%)
**Age; Median [Q1 - Q3]**	33 [31-35]	33 [31-36]
**Surgical vs. Non-Surgical; N (%)**		
Nonsurgical	7298 (78.1%)	2320 (80.9%)
Surgical	2042 (21.9%)	549 (19.1%)

© 2023 Han M et al. *JAMA Network Open.*

eTable 1. Year 1 PARS Score Category

Characteristic	Year 1 PARS Score Category				
	0 (N = 7001)	1-20 (N = 2023)	21+(N = 316)	Overall (N = 9340)	P-Value
**Age; Median (Q1-Q3)**	33 (31-35)	32 (31-35)	33 (31-35)	33 (31-35)	.02
**Clinician Sex; N (%)**					.21
Female	3400 (48.6%)	952 (47.1%)	164 (51.9%)	4516 (48.4%)	
Male	3597 (51.4%)	1071 (52.9%)	152 (48.1%)	4820 (51.6%)	
Missing	4 (0.1%)	0 (0%)	0 (0%)	4 (0.0%)	
**Year of Training; N (%)**					.39
2015	131 (1.9%)	38 (1.9%)	10 (3.2%)	179 (1.9%)	
2016	1652 (23.6%)	460 (22.7%)	80 (25.3%)	2192 (23.5%)	
2017	1715 (24.5%)	528 (26.1%)	86 (27.2%)	2329 (24.9%)	
2018	1854 (26.5%)	539 (26.6%)	74 (23.4%)	2467 (26.4%)	
2019	1649 (23.6%)	458 (22.6%)	66 (20.9%)	2173 (23.3%)	
**Program Region; N (%)**					<.001
Midwest	1909 (27.3%)	578 (28.6%)	97 (30.7%)	2584 (27.7%)	
Northeast	1408 (20.1%)	422 (20.9%)	80 (25.3%)	1910 (20.4%)	
Puerto Rico	3 (0.0%)	0 (0%)	0 (0%)	3 (0.0%)	

© 2023 Han M et al. *JAMA Network Open.*

Characteristic	Year 1 PARS Score Category				
	0 (N = 7001)	1-20 (N = 2023)	21+(N = 316)	Overall (N = 9340)	P-Value
South	1687 (24.1%)	547 (27.0%)	79 (25.0%)	2313 (24.8%)	
West	1994 (28.5%)	476 (23.5%)	60 (19.0%)	2530 (27.1%)	
**PARS Site Region; N (%)**					<.001
Midwest	2176 (31.1%)	665 (32.9%)	122 (38.6%)	2963 (31.7%)	
Northeast	628 (9.0%)	266 (13.1%)	33 (10.4%)	927 (9.9%)	
South	1601 (22.9%)	494 (24.4%)	79 (25.0%)	2174 (23.3%)	
West	2596 (37.1%)	598 (29.6%)	82 (25.9%)	3276 (35.1%)	
**US vs. Non-US Medical School; N (%)**					<.001
Non-US Med. School	1217 (17.4%)	425 (21.0%)	70 (22.2%)	1712 (18.3%)	
US Med. School	5784 (82.6%)	1598 (79.0%)	246 (77.8%)	7628 (81.7%)	
**Academic vs Non-Academic Practice Setting; N (%)**					<.001
Academic	4845 (69.2%)	1220 (60.3%)	165 (52.2%)	6230 (66.7%)	
Non-academic	2156 (30.8%)	803 (39.7%)	151 (47.8%)	3110 (33.3%)	

© 2023 Han M et al. *JAMA Network Open.*

Characteristic	Year 1 PARS Score Category				
	**0 (N = 7001)**	**1-20 (N = 2023)**	**21+(N = 316)**	**Overall (N = 9340)**	**P-Value**
**Surgical vs. Non-Surgical Clinician; N (%)**					<.001
Nonsurgical	5404 (77.2%)	1618 (80.0%)	276 (87.3%)	7298 (78.1%)	
Surgical	1597 (22.8%)	405 (20.0%)	40 (12.7%)	2042 (21.9%)	

Footnotes: P-values calculated using a Mann-Whitney U test for continuous variables and Pearson's chi-square test for categorical variables.

© 2023 Han M et al. *JAMA Network Open.*

eTable 2. Cumulative Link Mixed-Effects Model Testing Association of Minimum Professionalism/Communication Milestone Category With Year 1 PARS Category

Variable	Coefficient	Standard Error	P-value
Age	-0.014994	0.007086	0.034340
Male Gender (Reference = Female)	0.024294	0.050300	0.629120
2016 Training Year (Reference = 2015)	-0.047535	0.184217	0.796380
2017 Training Year (Reference = 2015)	0.056943	0.183550	0.756390
2018 Training Year (Reference = 2015)	-0.049916	0.183554	0.785670
2019 Training Year (Reference = 2015)	-0.095152	0.184709	0.606450
Non-US medical school (Reference = US medical school)	-0.022498	0.067303	0.738170
Non-Academic Practice Setting (Reference = Academic)	0.654464	0.223798	0.003450
Surgical Specialty (Reference = Non-Surgical)	-0.210165	0.064129	0.001050
Program Size Decile	-0.050229	0.010113	0.000001
Minimum Professionalism/Communication Milestone Category = 0 - 2.5 (Reference = 4.0)	0.205565	0.095188	0.030810
Minimum Professionalism/Communication Milestone Category = 3.0 - 3.5 (Reference = 4.0)	0.172895	0.055727	0.001920
Minimum Professionalism/Communication Milestone Category = 4.5 - 5.0 (Reference = 4.0)	-0.069465	0.084537	0.411240

Footnotes: Coefficients and standard errors derived using cumulative link mixed effects model (ordinal regression models with mixed effects) with outcome of PARS Year 1 Index Score Category (categories used were 0, 1-20, and 21+). Coefficients show log-odds of clinicians being included in a higher PARS Year 1 Index Score category compared those in shown reference category. Model includes a random effect for PARS site.

© 2023 Han M et al. *JAMA Network Open.*

eTable 3. Cumulative Link Mixed-Effects Model Testing Association of Lowest Professionalism Milestone Category With PARS Year 1 Index Score Category

Variable	Coefficient	Standard Error	P-value
Age	-0.01470	0.00709	0.03804
Male Gender (Reference = Female)	0.02421	0.05029	0.63023
2016 Training Year (Reference = 2015)	-0.04492	0.18419	0.80731
2017 Training Year (Reference = 2015)	0.05839	0.18353	0.75038
2018 Training Year (Reference = 2015)	-0.05019	0.18352	0.78450
2019 Training Year (Reference = 2015)	-0.09459	0.18470	0.60858
Non-US medical school (Reference = US medical school)	-0.02497	0.06728	0.71053
Non-Academic Practice Setting (Reference = Academic)	0.65360	0.22385	0.00350
Surgical Specialty (Reference = Non-Surgical)	-0.21293	0.06409	0.00089
Program Size Decile	-0.05019	0.01011	0.00000
Minimum Professionalism Milestone Category = 0 - 2.5 (Reference = 4.0)	0.15334	0.10486	0.14366
Minimum Professionalism Milestone Category = 3.0 - 3.5 (Reference = 4.0)	0.12870	0.05621	0.02204
Minimum Professionalism Milestone Category = 4.5 - 5.0 (Reference = 4.0)	-0.10767	0.07458	0.14883

Footnotes: Coefficients and standard errors derived using cumulative link mixed effects model (ordinal regression models with mixed effects) with outcome of PARS Year 1 Index Score Category (categories used were 0, 1-20, and 21+). Coefficients show log-odds of clinicians being included in a higher PARS Year 1 Index Score category compared those in shown reference category. Model includes a random effect for PARS site.

© 2023 Han M et al. *JAMA Network Open.*

eTable 4. Cumulative Link Mixed-Effects Model Testing Association of Lowest Communication Milestone Category With PARS Year 1 Index Score Category

Variable	Coefficient	Standard Error	P-value
Age	-0.015452	0.007089	0.029290
Male Gender (Reference = Female)	0.023490	0.050308	0.640560
2016 Training Year (Reference = 2015)	-0.051225	0.184261	0.781010
2017 Training Year (Reference = 2015)	0.054027	0.183586	0.768540
2018 Training Year (Reference = 2015)	-0.055415	0.183583	0.762770
2019 Training Year (Reference = 2015)	-0.100636	0.184741	0.585930
Non-US medical school (Reference = US medical school)	-0.020897	0.067333	0.756300
Non-Academic Practice Setting (Reference = Academic)	0.653490	0.224000	0.003530
Surgical Specialty (Reference = Non-Surgical)	-0.212618	0.064160	0.000920
Program Size Decile	-0.050154	0.010112	0.000001
Minimum Communication Milestone Category = 0 - 2.5 (Reference = 4.0)	0.205394	0.113736	0.070940
Minimum Communication Milestone Category = 3.0 - 3.5 (Reference = 4.0)	0.115607	0.055832	0.038390
Minimum Communication Milestone Category = 4.5 - 5.0 (Reference = 4.0)	-0.127439	0.075041	0.089460

Footnotes: Coefficients and standard errors derived using cumulative link mixed effects model (ordinal regression models with mixed effects) with outcome of PARS Year 1 Index Score Category (categories used were 0, 1-20, and 21+). Coefficients show log-odds of clinicians being included in a higher PARS Year 1 Index Score category compared those in shown reference category. Model includes a random effect for PARS site.

© 2023 Han M et al. *JAMA Network Open.*

eTable 5. Cumulative Link Mixed-Effects Model Testing Association of Lowest Patient Care Milestone Category With PARS Year 1 Index Score Category

Variable	Coefficient	Standard Error	P-value
Age	-0.0154610	0.0070940	0.0292980
Male Gender (Reference = Female)	0.0238990	0.0503180	0.6348220
2016 Training Year (Reference = 2015)	-0.0549460	0.1842520	0.7655430
2017 Training Year (Reference = 2015)	0.0510700	0.1836220	0.7809150
2018 Training Year (Reference = 2015)	-0.0613500	0.1836190	0.7382910
2019 Training Year (Reference = 2015)	-0.1068870	0.1848220	0.5630440
Non-US medical school (Reference = US medical school)	-0.0194420	0.0673490	0.7728270
Non-Academic Practice Setting (Reference = Academic)	0.6489470	0.2232960	0.0036580
Surgical Specialty (Reference = Non-Surgical)	-0.2401450	0.0636560	0.0001620
Program Size Decile	-0.0492090	0.0101160	0.0000012
Minimum Patient Care Milestone Category = 0 - 2.5 (Reference = 4.0)	0.2633230	0.0934650	0.0048420
Minimum Patient Care Milestone Category = 3.0 - 3.5 (Reference = 4.0)	0.2211960	0.0570350	0.0001050
Minimum Patient Care Milestone Category = 4.5 - 5.0 (Reference = 4.0)	0.0016100	0.1015910	0.9873530

Footnotes: Coefficients and standard errors derived using cumulative link mixed effects model (ordinal regression models with mixed effects) with outcome of PARS Year 1 Index Score Category (categories used were 0, 1-20, and 21+). Coefficients show log-odds of clinicians being included in a higher PARS Year 1 Index Score category compared those in shown reference category. Model includes a random effect for PARS site.

© 2023 Han M et al. *JAMA Network Open.*

eTable 6. Cumulative Link Mixed-Effects Model Testing Association of Lowest Medical Knowledge Milestone Category With PARS Year 1 Index Score Category

Variable	Coefficient	Standard Error	P-value
Age	-0.0157450	0.0071000	0.0265850
Male Gender (Reference = Female)	0.0287770	0.0502980	0.5672280
2016 Training Year (Reference = 2015)	-0.0623370	0.1842410	0.7351020
2017 Training Year (Reference = 2015)	0.0399070	0.1835620	0.8278950
2018 Training Year (Reference = 2015)	-0.0692930	0.1835870	0.7058470
2019 Training Year (Reference = 2015)	-0.1161150	0.1847360	0.5296450
Non-US medical school (Reference = US medical school)	-0.0235540	0.0672680	0.7262230
Non-Academic Practice Setting (Reference = Academic)	0.6512390	0.2242600	0.0036850
Surgical Specialty (Reference = Non-Surgical)	-0.2382790	0.0635880	0.0001790
Program Size Decile	-0.0504680	0.0101090	0.0000006
Minimum Medical Knowledge Milestone Category = 0 - 2.5 (Reference = 4.0)	0.1026400	0.0914620	0.2617740
Minimum Medical Knowledge Milestone Category = 3.0 - 3.5 (Reference = 4.0)	0.1665850	0.0561220	0.0029950
Minimum Medical Knowledge Milestone Category = 4.5 - 5.0 (Reference = 4.0)	-0.0055030	0.0889010	0.9506420

Footnotes: Coefficients and standard errors derived using cumulative link mixed effects model (ordinal regression models with mixed effects) with outcome of PARS Year 1 Index Score Category (categories used were 0, 1-20, and 21+). Coefficients show log-odds of clinicians being included in a higher PARS Year 1 Index Score category compared those in shown reference category. Model includes a random effect for PARS site.

© 2023 Han M et al. *JAMA Network Open.*

eTable 7. Sensitivity Analysis: Ordinal Regression Model Treating Program as a Fixed Effect

Variable	Coefficient	Standard Error	P-value
Age	-0.0123	0.007	0.0795
Male Gender (Reference = Female)	-0.0491	0.0511	0.337
2016 Training Year (Reference = 2015)	-0.0837	0.182	0.6457
2017 Training Year (Reference = 2015)	0.0226	0.1812	0.9009
2018 Training Year (Reference = 2015)	-0.1056	0.1814	0.5603
2019 Training Year (Reference = 2015)	-0.1014	0.1824	0.5784
PARS Region = Northeast (Reference = Midwest)	0.3527	0.084	<0.0001
PARS Region = South (Reference = Midwest)	0.3231	0.0752	<0.0001
PARS Region = West (Reference = Midwest)	0.0474	0.0715	0.5068
Non-US medical school (Reference = US medical school)	-0.0916	0.0665	0.1685
Non-Academic Practice Setting (Reference = Academic)	0.3185	0.063	<0.0001
CT Surgery Program (Reference = Anesthesiology)	0.9736	0.4499	0.0304
Emergency Medicine Program (Reference = Anesthesiology)	2.1099	0.1686	<0.0001
General Surgery Program (Reference = Anesthesiology)	1.4861	0.1915	<0.0001
Genitourinary Surgery Program (Reference = Anesthesiology)	0.8109	0.1832	<0.0001
Head & Neck Surgery Program (Reference = Anesthesiology)	0.9712	0.2393	<0.0001
Internal Medicine Program (Reference = Anesthesiology)	1.2176	0.1537	<0.0001
Orthopedics - Surgical Program (Reference = Anesthesiology)	0.7575	0.1992	0.0001
Other Non-Surgical Program (Reference = Anesthesiology)	0.9543	0.1704	<0.0001
Other Surgical Program (Reference = Anesthesiology)	0.5143	0.2023	0.011
Pediatrics - Anesthesiology Program (Reference = Anesthesiology)	0.3159	0.3452	0.3601
Pediatrics - General Program (Reference = Anesthesiology)	0.4725	0.1915	0.0136
Pediatrics - Internal Medicine Program (Reference = Anesthesiology)	0.6551	0.2038	0.0013
Pediatrics - Other Non-Surgical Program (Reference = Anesthesiology)	0.8035	0.2727	0.0032
Pediatrics - Radiology Program (Reference = Anesthesiology)	-7.4575	41.9657	0.859

© 2023 Han M et al. *JAMA Network Open.*

Variable	Coefficient	Standard Error	P-Value
Plastic Surgery Program (Reference = Anesthesiology)	0.8429	0.3333	0.0114
Radiology Program (Reference = Anesthesiology)	-0.4935	0.2919	0.0909
Vascular Surgery Program (Reference = Anesthesiology)	1.734	0.3339	<0.0001
Program Size Decile	-0.0453	0.0099	<0.0001
Minimum Professionalism/Communication Milestone Category = 0 - 2.5 (Reference = 4.0)	0.2428	0.0955	0.011
Minimum Professionalism/Communication Milestone Category = 3.0 - 3.5 (Reference = 4.0)	0.1632	0.0553	0.0032
Minimum Professionalism/Communication Milestone Category = 4.5 - 5.0 (Reference = 4.0)	-0.1068	0.084	0.2036

Footnotes: Coefficients and standard errors derived using ordinal regression model with outcome of PARS Year 1 Index Score Category (categories used were 0, 1-20, and 21+). Coefficients show log-odds of clinicians being included in a higher PARS Year 1 Index Score category compared to shown reference category.

© 2023 Han M et al. *JAMA Network Open.*

eTable 8. Sensitivity Analysis: Cumulative Link Mixed-Effects Model Clustering on Residency Site

Variable	Coefficient	Standard Error	P-value
Age	-0.011385	0.006956	0.101684
Male Gender (Reference = Female)	-0.011815	0.049349	0.810782
2016 Training Year (Reference = 2015)	-0.071615	0.180242	0.691125
2017 Training Year (Reference = 2015)	0.023728	0.179547	0.894862
2018 Training Year (Reference = 2015)	-0.065818	0.179565	0.713959
2019 Training Year (Reference = 2015)	-0.100724	0.180699	0.577244
Non-US medical school (Reference = US medical school)	-0.057367	0.066205	0.386220
Non-Academic Practice Setting (Reference = Academic)	0.288563	0.057823	0.000001
Surgical Specialty (Reference = Non-Surgical)	-0.227745	0.063440	0.000331
Program Size Decile	-0.056335	0.012391	0.000005
Minimum Professionalism/Communication Milestone Category = 0 - 2.5 (Reference = 4.0)	0.199349	0.093952	0.033854
Minimum Professionalism/Communication Milestone Category = 3.0 - 3.5 (Reference = 4.0)	0.171014	0.054811	0.001808
Minimum Professionalism/Communication Milestone Category = 4.5 - 5.0 (Reference = 4.0)	-0.058238	0.083175	0.483810

Footnotes: Coefficients and standard errors derived using cumulative link mixed effects model (ordinal regression models with mixed effects) with outcome of PARS Year 1 Index Score Category (categories used were 0, 1-20, and 21+). Coefficients show log-odds of clinicians being included in a higher PARS Year 1 Index Score category compared those in shown reference category. Model includes a random effect for residency site.

© 2023 Han M et al. *JAMA Network Open.*

eTable 9. Sensitivity Analysis: Cumulative Link Mixed-Effects Model Clustering on Training Program

Variable	Coefficient	Standard Error	P-value
Age	-0.0129640	0.0069950	0.0638400
Male Gender (Reference = Female)	-0.0469130	0.0508850	0.3565600
2016 Training Year (Reference = 2015)	-0.0851490	0.1814510	0.6388800
2017 Training Year (Reference = 2015)	0.0153810	0.1807050	0.9321700
2018 Training Year (Reference = 2015)	-0.1095860	0.1808240	0.5444900
2019 Training Year (Reference = 2015)	-0.1153260	0.1818350	0.5259300
Non-US medical school (Reference = US medical school)	-0.0964760	0.0659210	0.1433300
Non-Academic Practice Setting (Reference = Academic)	0.2592610	0.0550380	0.0000025
Surgical Specialty (Reference = Non-Surgical)	-0.0659470	0.2447460	0.7875800
Program Size Decile	-0.0436840	0.0098000	0.0000083
Minimum Professionalism/Communication Milestone Category = 0 - 2.5 (Reference = 4.0)	0.2332420	0.0951700	0.0142500
Minimum Professionalism/Communication Milestone Category = 3.0 - 3.5 (Reference = 4.0)	0.1555110	0.0550780	0.0047500
Minimum Professionalism/Communication Milestone Category = 4.5 - 5.0 (Reference = 4.0)	-0.0941370	0.0837280	0.2608800

Footnotes: Coefficients and standard errors derived using cumulative link mixed effects model (ordinal regression models with mixed effects) with outcome of PARS Year 1 Index Score Category (categories used were 0, 1-20, and 21+). Coefficients show log-odds of clinicians being included in a higher PARS Year 1 Index Score category compared those in shown reference category. Model includes a random effect for training program.

© 2023 Han M et al. *JAMA Network Open.*

eTable 10. Sensitivity Analysis: Cumulative Link Mixed-Effects Model Clustering on Clinician Specialty

Variable	Coefficient	Standard Error	P-value
Age	-0.002287	0.007355	0.755859
Male Gender (Reference = Female)	-0.051680	0.052955	0.329104
2016 Training Year (Reference = 2015)	0.029875	0.184633	0.871458
2017 Training Year (Reference = 2015)	0.121479	0.183969	0.509046
2018 Training Year (Reference = 2015)	-0.008882	0.184021	0.961506
2019 Training Year (Reference = 2015)	-0.018134	0.184955	0.921895
Non-US medical school (Reference = US medical school)	-0.133568	0.068558	0.051386
Non-Academic Practice Setting (Reference = Academic)	0.254816	0.056588	0.000007
Surgical Specialty (Reference = Non-Surgical)	0.175727	0.158800	0.268471
Program Size Decile	-0.037066	0.010442	0.000386
Minimum Professionalism/Communication Milestone Category = 0 - 2.5 (Reference = 4.0)	0.191174	0.099165	0.053875
Minimum Professionalism/Communication Milestone Category = 3.0 - 3.5 (Reference = 4.0)	0.124352	0.057130	0.029508
Minimum Professionalism/Communication Milestone Category = 4.5 - 5.0 (Reference = 4.0)	0.001163	0.086253	0.989239

Footnotes: Coefficients and standard errors derived using cumulative link mixed effects model (ordinal regression models with mixed effects) with outcome of PARS Year 1 Index Score Category (categories used were 0, 1-20, and 21+). Coefficients show log-odds of clinicians being included in a higher PARS Year 1 Index Score category compared those in shown reference category. Model includes a random effect for clinician specialty.

© 2023 Han M et al. *JAMA Network Open.*

eTable 11. Sensitivity Analysis: Cumulative Link Mixed-Effects Model Clustering on Program Size Decile

Variable	Coefficient	Standard Error	P-value
Age	-0.011801	0.006851	0.084970
Male Gender (Reference = Female)	0.000918	0.048666	0.984950
2016 Training Year (Reference = 2015)	-0.074321	0.177989	0.676270
2017 Training Year (Reference = 2015)	0.032494	0.177276	0.854570
2018 Training Year (Reference = 2015)	-0.056634	0.177319	0.749430
2019 Training Year (Reference = 2015)	-0.092778	0.178387	0.603000
Non-US medical school (Reference = US medical school)	-0.071043	0.064344	0.269540
Non-Academic Practice Setting (Reference = Academic)	0.304962	0.054183	0.000000
Surgical Specialty (Reference = Non-Surgical)	-0.216526	0.062008	0.000480
Minimum Professionalism/Communication Milestone Category = 0 - 2.5 (Reference = 4.0)	0.204633	0.092726	0.027320
Minimum Professionalism/Communication Milestone Category = 3.0 - 3.5 (Reference = 4.0)	0.155310	0.053991	0.004020
Minimum Professionalism/Communication Milestone Category = 4.5 - 5.0 (Reference = 4.0)	-0.068499	0.082226	0.404810

Footnotes: Coefficients and standard errors derived using cumulative link mixed effects model (ordinal regression models with mixed effects) with outcome of PARS Year 1 Index Score Category (categories used were 0, 1-20, and 21+). Coefficients show log-odds of clinicians being included in a higher PARS Year 1 Index Score category compared those in shown reference category. Model includes a random effect for program size decile.

© 2023 Han M et al. *JAMA Network Open.*
