# Supplementary material for: Trainee Physician Milestone Ratings and Patient Complaints in Early Posttraining Practice
Source: JAMA Netw Open. 2023 Apr 11;6(4):e237588. doi: 10.1001/jamanetworkopen.2023.7588 (PMC10091163; doi:10.1001/jamanetworkopen.2023.7588)
Supplement: Supplement 2. — Data Sharing Statement [file jamanetwopen-e237588-s002.pdf]

Data Sharing Statement

Han. Trainee Physician Milestone Ratings and Patient Complaints in Early Posttraining Practice. *JAMA Netw Open*. Published April 11, 2023.
doi:10.1001/jamanetworkopen.2023.7588

Data
Data available: No
